# Electrical injury – a dual center analysis of patient characteristics, therapeutic specifics and outcome predictors

**DOI:** 10.1186/s13049-018-0513-2

**Published:** 2018-05-31

**Authors:** Jochen Gille, Thomas Schmidt, Adrian Dragu, Dimitri Emich, Peter Hilbert-Carius, Thomas Kremer, Thomas Raff, Beate Reichelt, Apostolos Siafliakis, Frank Siemers, Michael Steen, Manuel F. Struck

**Affiliations:** 1 Department of Anesthesiology, Intensive Care Medicine and Pain Therapy, St. Georg Hospital, Delitzscher Str. 141, 04129 Leipzig, Germany; 2 Department of Medical Psychology, Bergmannstrost Hospital, Merseburger Str. 165, 06112 Halle, Germany; 3 Department of Plastic and Hand Surgery, Burn Center, St. Georg Hospital, Delitzscher Str. 141, 04129 Leipzig, Germany; 4 Department of Plastic and Hand Surgery, University Center of Orthopedics and Trauma Surgery, University Hospital Carl Gustav Carus, Fetscherstraße 74, 01307 Dresden, Germany; 5 Department of Anesthesiology, Intensive Care and Emergency Medicine, Bergmannstrost Hospital, Merseburger Str. 165, 06112 Halle, Germany; 6 Department of Plastic and Hand Surgery, Burn Center, Bergmannstrost Hospital, Merseburger Str. 165, 06112 Halle, Germany; 7 Department of Anesthesiology and Intensive Care Medicine, University Hospital Leipzig, Liebigstr. 20, 04103 Leipzig, Germany

**Keywords:** Electrical injury, Burns, High voltage, Creatinine kinase, Myoglobin, Amputation

## Abstract

**Background:**

Electrical injuries represent life-threatening emergencies. Evidence on differences between high (HVI) and low voltage injuries (LVI) regarding characteristics at presentation, rhabdomyolysis markers, surgical and intensive burn care and outcomes is scarce.

**Methods:**

Consecutive patients admitted to two burn centers for electrical injuries over an 18-year period (1998–2015) were evaluated. Analysis included comparisons of HVI vs. LVI regarding demographic data, diagnostic and treatment specific variables, particularly serum creatinine kinase (CK) and myoglobin levels over the course of 4 post injury days (PID), and outcomes.

**Results:**

Of 4075 patients, 162 patients (3.9%) with electrical injury were analyzed. A total of 82 patients (50.6%) were observed with HVI. These patients were younger, had considerably higher morbidity and mortality, and required more extensive burn surgery and more complex burn intensive care than patients with LVI. Admission CK and myoglobin levels correlated significantly with HVI, burn size, ventilator days, surgical interventions, amputation, flap surgery, renal replacement therapy, sepsis, and mortality. The highest serum levels were observed at PID 1 (myoglobin) and PID 2 (CK). In 23 patients (14.2%), cardiac arrhythmias were observed; only 4 of these arrhythmias occurred after hospital admission. The independent predictors of mortality were ventilator days (OR 1.27, 95% CI 1.06–1.51, *p* = 0.009), number of surgical interventions (OR 0.47, 95% CI 0.27–0.834, *p* = 0.010) and limb amputations (OR 14.26, 95% CI 1.26–162.1, *p* = 0.032).

**Conclusions:**

Patients with electrical injuries, HVI in particular, are at high risk for severe complications. Due to the need for highly specialized surgery and intensive care, treatment should be reserved to burn units. Serum myoglobin and CK levels reflect the severity of injury and may predict a more complex clinical course. Routine cardiac monitoring > 24 h post injury does not seem to be necessary.

## Background

Electrical injuries are rare but potentially life-threatening emergencies. Voltage exposure is defined by industrial norms as either above or below 1000 V (high voltage injuries (HVI) or low voltage injuries (LVI), respectively) [1]. The cutoff value of 1000 V is not supported by clinical data, and LVI may cause similar damage to the human body as HVI, depending on individual current pathways, amperage and the duration of current exposure. Usually, HVI causes high temperatures in tissues of greater electrical resistance, leading to extensive deep tissue injuries and microvascular coagulation, whereas LVI causes less invasive tissue lesions but is more likely to induce malignant cardiac arrhythmia and higher rates of neurologic long-term sequelae [1–8]. HVI and LVI may both account for complex neurovascular lesions. Clinical symptoms may be considerably delayed and often require repetitive surgery. Additionally, extensive muscular necrosis and rhabdomyolysis may lead to acute kidney injury (AKI), coagulation disorders, compartment syndrome and amputation [1–3]. The goal of this study was to explore differences in prehospital care, admission characteristics, burn intensive care, surgery and outcomes in patients requiring admission to a burn intensive care unit (BICU) after HVI and LVI.

## Methods

After approval of the local ethics committees, the databases of two neighboring burn centers in Central Germany were reviewed in order to identify patients who were admitted to the BICU for the treatment of electrical injuries between 01/1998 and 12/2015. Electrical injuries were defined as direct electrical injuries, electric arc burns and concomitant burns due to HVI or LVI. Both centers were comparable regarding infrastructure, staff and adherence to national recommendations. The catchment areas of both centers are coordinated by the regional emergency dispatching services and by the national burn depository of the fire dispatching center of the City of Hamburg. The distance of the two burn centers is 40 km and together, they have a catchment area of 3 federal states involving approximately 8.5 million inhabitants. The burn center in Leipzig provides six BICU beds and six intermediate care beds whereas the burn center in Halle provides eight BICU beds.

### General management

Patients were admitted to the burn resuscitation room from the trauma scene or were secondarily transferred from other hospitals. The indications for BICU admission comprised surgical burn wound management, potential risk for rhabdomyolysis and cardiac arrhythmia. Emergency measures included tracheal intubation in case of progressive airway edema or respiratory insufficiency, central venous and arterial access, urinary catheterization, electrocardiography and cardiac monitoring. If HVI compromised limb circulation, pulse oximetry was routinely applied to detect peripheral perfusion deficits. In cases of progressive edema and compartment syndrome, decompression escharotomy or fasciotomy was performed immediately. Fluid resuscitation was initiated using lactated or acetated Ringer’s solution according to Parkland formulas and target urinary outputs of 0.5 ml/kg/h. A more aggressive approach (estimating a urinary output of 2 ml/kg/h) was performed in the presence of relevant myoglobinemia (serum myoglobin level > 1000 μg/l). Application of diuretics was avoided within the first 24 h. Pharmacological strategies, such as application of mannitol or alkalization by adding sodium bicarbonate to the intravenous solution, were not performed routinely but were considered in cases with severe rhabdomyolysis (serum myoglobin > 3000 μg/l). Renal replacement therapy (RRT) was initiated only in manifest AKI using continuous veno-venous hemodiafiltration (CVVHDF) or continuous veno-venous hemodialysis (CVVHD). Additional vasopressors were used in order to maintain mean arterial blood pressures > 70 mmHg to provide tissue oxygenation and avoid extensive fluid creep. Necrosectomy and split thickness skin grafting were performed within 72 h after admission, depending on the patient’s individual condition. Limb amputation and microvascular tissue transplantation were performed in extensive muscle necrosis according to the treatment protocols of the burn centers.

### Statistics

Data are presented as the mean ± standard deviation and counts (percentage). Statistical comparisons between survivors and non-survivors were performed using the χ2 test for qualitative data and Student’s t test or Mann-Whitney U-test for quantitative data. The alpha level of significance (p) was set at 0.05. All tests were two-tailed. Univariate analysis was performed to identify possible predictors of mortality. Variables tested included demographic data, burned total body surface area (TBSA), emergency airway management, cardiac arrhythmia electrocardiography and time, admission temperature, serum myoglobin, creatine kinase (CK) and troponin levels over the course of four PID, intensive care, surgical procedures and outcomes. Logistic regression analysis was performed to identify independent predictors of mortality. Data were obtained from paper-based and electronic charts.

## Results

**Fig. 1:**
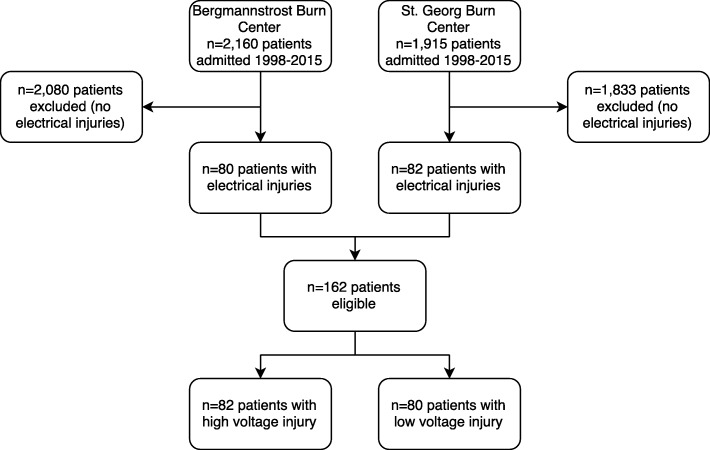
Study flow chart

During the study period, 4075 patients were admitted to the two BICUs; 162 (3.9%) of these patients met the inclusion criteria and thus were subject of the study (Fig. 1). Of the 162 patients, 11 patients (6.8%) were aged below 18 years (range 12–17 years). HVI was present in 82 patients (50.6%) (Tables 1, 2 and 3). Most patients were male (94.4%), and the mean age was 37.7 ± 15.5 years (Table 1). Emergency tracheal intubation was required in 74 patients (45.6%) (Table 2). The need for tracheal intubation was significantly associated with mortality (*p* < 0.001). Cardiac arrhythmia was observed in 23 patients (14.2%), including seven patients who required cardiopulmonary resuscitation (CPR) at the scene of whom five survived until BICU discharge to rehabilitation (Table 1). Four patients (2.5%) presented cardiac arrhythmias after hospital admission. All arrhythmias were self-limiting without compromising the patient. In one patient, arrhythmia attributable to the electric injury was observed > 24 h after admission. HVI patients required significantly more extensive burn intensive care and surgery than LVI patients (Table 2).

**Table 1:** Demography, clinical presentation, cardiac arrhythmia and trauma context of patients with electric injury

	Total (*n* = 162)	> = 1000 V (*n* = 82)	< 1000 V (*n* = 80)	*p* value
Age, years; mean (SD)	37.74 (15.52)	32.76 (15.19)	42.85 (14.21)	*< 0.001*
Male; n (%)	153 (94.4)	77 (93.9)	76 (95.0)	0.760
Clinical presentation				
% TBSA burned; mean (SD)	16.0 (18.77)	25.93 (21.34)	5.85 (6.67)	*< 0.001*
ABSI; mean (SD)	5.21 (2.29)	6.32 (2.44)	4.08 (1.40)	*< 0.001*
Inhalation injury; n (%)	13 (8.0)	10 (12.2)	3 (3.8)	*0.048*
Admission temperature, °C; mean (SD)	35.67 (1.32)	35.17 (1.59)	36.18 (.67)	*< 0.001*
Cardiac arrhythmia				0.209
On scene; n (%)	18 (11.1)	11 (13.4)	7 (8.8)	
EMS transport; n (%)	1 (.6)	0 (0.0)	1 (1.3)	
BICU < 24 h; n (%)	3 (1.9)	0 (0.0)	3 (3.8)	
BICU > 24 h; n (%)	1 (.6)	1 (1.2)	0 (0.0)	
				0.124
Extrasystole/ST/AF; n (%)	9 (5.6)	2 (2.4)	7 (8.8)	
VT/VF; n (%)	3 (1.9)	2 (2.4)	1 (1.3)	
Asystole/PEA; n (%)	4 (2.5)	4 (4.9)	0 (0.0)	
Trauma context; n (%)				*0.002*
Work	105 (64.8)	43 (52.4)	62 (77.5)	
Non-work	48 (29.6)	31 (24.3)	17 (23.7)	
Suicide attempt	9 (5.6)	8 (9.8)	1 (1.3)	

*SD* standard deviation, *TBSA* total body surface area, *ABSI* abbreviated burn severity index, *EMS* emergency medical service, *BICU* burn intensive care unit, *ST/AF* sinus tachycardia/atrial fibrillation, *VT/VF* ventricular tachycardia/ventricular fibrillation, *PEA* pulseless electric activity

italicized *p* values indicate statistical significance (*p* < 0.05)

**Table 2:** Emergency tracheal intubation, surgery, burn intensive care, and outcomes of patients with electric injury

	Total (*n* = 162)	> = 1000 V (*n* = 82)	< 1000 V (*n* = 80)	*p* value
Emergency tracheal Intubation				*< 0.001*
On scene EMS; n (%)	60 (37.0)	53 (64.6)	7 (8.8)	
Resuscitation room; n (%)	14 (8.6)	8 (9.8)	6 (7.5)	
Surgery				
Interventions; mean (SD)	2.77 (3.28)	4.07 (3.70)	1.44 (2.10)	*< 0.001*
Fasciotomy; n (%)	29 (17.9)	27 (32.9)	2 (2.5)	*< 0.001*
Split thickness skin graft; n (%)	116 (71.6)	71 (86.6)	45 (56.3)	*< 0.001*
Flap surgery; n (%)	24 (14.8)	18 (22.0)	6 (7.5)	*0.010*
Minor limb amputation; n (%)	23 (14.2)	21 (25.6)	2 (2.5)	*< 0.001*
Major limb amputation; n (%)	2 (1.2)	2 (2.4)	0 (0.0)	
Decompression laparotomy; n (%)	5 (3.1)	5 (6.1)	0 (0.0)	*0.025*
Burn intensive care				
Mechanical ventilation; n (%)	71 (43.8)	59 (72.0)	12 (15.0)	*< 0.001*
Ventilator days; mean (SD)	3.89 (8.98)	6.83 (10.96)	0.88 (4.79)	*< 0.001*
Prone positioning/Rotorest®; n (%)	13 (8.0)	12 (14.6)	1 (1.3)	*0.002*
Renal replacement therapy; n (%)	18 (11.1)	17 (20.7)	1 (1.3)	*< 0.001*
Sepsis; n (%)	31 (19.1)	26 (31.7)	5 (6.3)	*< 0.001*
Outcome				
LOS BICU, days; mean (SD)	20.98 (20.54)	29.49 (22.62)	12.25 (13.52)	*< 0.001*
30-day mortality; n (%)	11 (6.8)	11 (13.4)	0 (0.0)	*< 0.001*
Hospital mortality; n (%)	17 (10.5)	15 (18.3)	2 (2.5)	*< 0.001*

*SD* standard deviation, *EMS* emergency medical service, *LOS* length of stay, *BICU* burn intensive care unit

italicized *p* values indicate statistical significance (*p* < 0.05)

**Table 3:** Admission characteristics of patients with electrical injury

	Total (*n* = 162)	> = 1000 V (*n* = 82)	< 1000 V (*n* = 80)	*p* value
BICU admission; n (%)				0.950
From scene	111 (68.5)	56 (68.3)	55 (68.8)	
Interfacility	51 (31.5)	26 (31.7)	25 (31.3)	
EMS vehicle; n (%)				*< 0.001*
Ground ambulance	92 (56.8)	34 (41.5)	58 (72.5)	
Helicopter rescue	70 (43.2)	48 (58.5)	22 (27.5)	
BICU admission time after injury; n (%)				0.774
< 1 h	29 (17.9)	16 (19.5)	13 (16.3)	
1-4 h	107 (66.0)	53 (64.6)	54 (67.5)	
4-8 h	18 (11.1)	10 (12.2)	8 (10.0)	
8-24 h	4 (2.5)	2 (2.4)	2 (2.5)	
24-48 h	2 (1.2)	1 (1.2)	1 (1.3)	
> 48 h	2 (1.2)	0 (0.0)	2 (2.5)	
BICU distance to scene, km; mean (SD)	78.08 (62.29)	83.67 (62.29)	72.36 (71.02)	*< 0.001*

*BICU* burn intensive care unit, *EMS* Emergency medical service

italicized *p* values indicate statistical significance (*p* < 0.05)

**Fig. 2:**
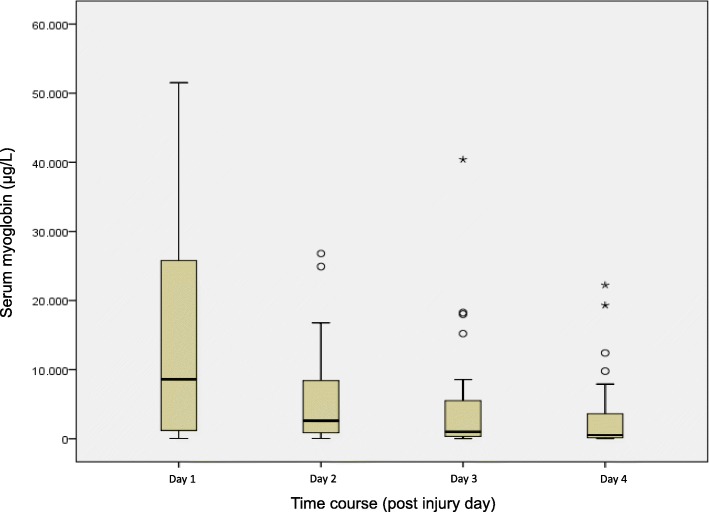
Boxplots of serum myoglobin values of high voltage injury patients. In the boxes, the dark horizontal line represents the median, with the box representing the 25th and 75th percentiles, the whiskers the 5th and 95th percentiles, the circles the outliers, and extreme outliers (three times the height of the boxes) represented by asterisks

**Fig. 3:**
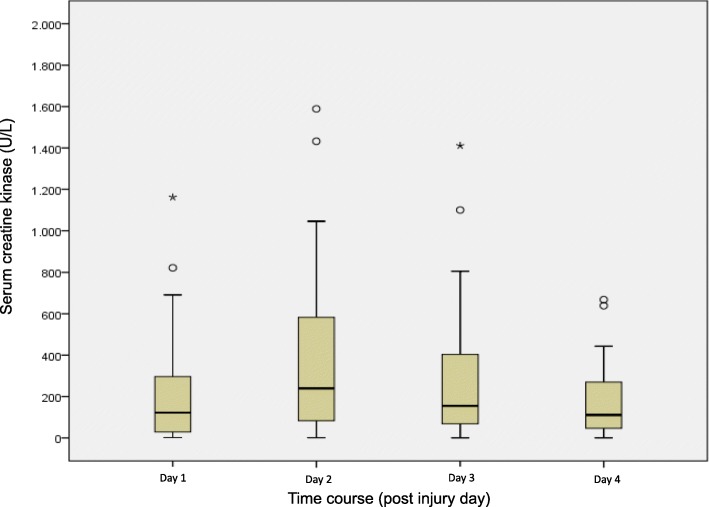
Boxplots of serum creatine kinase values of high voltage injury patients. In the boxes, the dark horizontal line represents the median, with the box representing the 25th and 75th percentiles, the whiskers the 5th and 95th percentiles, the circles the outliers, and extreme outliers (three times the height of the boxes) represented by asterisks

Admission serum myoglobin and CK levels were significantly associated with HVI, TBSA, BICU length of stay (LOS), ventilator days, number of interventions, mortality, amputation, flap surgery, RRT, and sepsis (*p* < 0.001). In HVI, the highest serum levels were measured at PID 1 (myoglobin) and PID 2 and 3 (CK) (Figs. 2 and 3). Troponin values were associated with HVI, mortality, flap surgery, RRT and sepsis but not with limb amputation or cardiac arrhythmia.

HVI patients required significantly longer BICU LOS, more ventilator days and had a higher incidence of sepsis and RRT and significantly increased 30-day and hospital mortality (*p* < 0.001) than LVI (Table 2). Logistic regression analysis identified ventilator days (OR 1.27, 95% CI 1.06–1.51, *p* = 0.009), number of surgical interventions (OR 0.47, 95% CI 0.27–0.834, *p* = 0.010) and limb amputations (OR 14.26, 95% CI 1.26–162.1, *p* = 0.032) as independent predictors of mortality of HVI and LVI (Table 4).

**Table 4:** Multivariate analysis for mortality/independent predictors

Parameter	OR	Lower 95% CI	Upper 95% CI	*p* value
Cardiac arrhythmia	2.25	0.24	21.20	0.479
Voltage (kV)	0.33	0.02	6.92	0.477
Intubation on scene	3.56	0.22	57.16	0.370
Ventilator days	1.27	1.06	1.51	*0.009*
Age (years)	1.05	0.95	1.15	0.330
Admission temperature (°C)	0.99	0.51	1.95	0.990
ABSI score (points)	1.13	0.42	3.00	0.812
TBSA burned (%)	1.11	0.98	1.27	0.111
Interventions	0.47	0.27	0.83	*0.010*
Limb amputation	14.26	1.26	162.1	*0.032*

*ABSI* abbreviated burn severity index, *TBSA* total body surface area, *OR* odds ratio, *CI* confidence interval

italicized *p* values indicate statistical significance (*p* < 0.05)

## Discussion

In our dual center study, electrical injuries accounted for a relatively low 3.9% proportion of BICU admissions, which is in line with the results of a recent literature review that estimated an incidence of below 5% [1]. The results of our study show that patients suffering from HVI show significantly higher morbidity and mortality rates than low voltage victims. Patients with HVI required more extensive burn surgery and more complex burn intensive care, which confirms the findings of previous studies [1–3, 7, 9]. Independent predictors of mortality of electrical injury (both HVI and LVI) were respirator days, number of surgical interventions, and incidence of amputation. In contrast to our results, other studies observed even higher mortality rates in LVI patients than in patients with high voltage trauma. This discrepancy may be explained by the fact that these studies included patients with fatal outcomes at the accident site, which may be related to initial cardiac arrhythmia and subsequent arrest. Consequently, LVI should not be under-estimated [1].

In the literature, most electrical injuries were observed in work-related accidents [1, 2, 4, 7]. This finding was also observed in our study, where two-thirds of the patients had work-related injuries. Interestingly, non-work-related injuries had a significantly higher proportion of HVI  (Table 1). This subgroup of patients included mainly younger patients who had accidents at railway areas (“train surfing” and other illegal activities). Studies of acute prehospital emergency approaches to patients with electrical injuries are scarce. We observed that patients suffering from HVI were more frequently admitted via helicopter rescue (Table 3), which was probably due to longer distances to the burn centers and more complex injury patterns (higher rates of tracheal intubation at the scene); however, the incidence of prehospital cardiac arrhythmia in HVI patients was comparable to that of LVI patients. Cardiac arrhythmia after BICU admission was observed in only 2.5% of our patients, all of which were self-limiting and did not require intervention. This result may support previous studies, which suggested that prolonged cardiac monitoring after electrical injuries, particularly LVI, is not required [8, 10, 11]. Moreover, other studies found that left ventricular dysfunction and cardiac injury were uncommon and not associated with serum troponin and CK in patients after HVI [12, 13]. A new marker of cardiac injury after electrical injury might be brain natriuretic peptide (BNP), whereas prospective studies regarding the diagnostic accuracy and independence of confounding factors are currently not available [14].

Particularly in HVI, rhabdomyolysis markers, such as serum myoglobin and CK, were associated with injury severity, RRT rate, amputation rate and mortality, as published previously [1–3, 15–19]. The highest myoglobin level was observed at BICU admission with a linear decrease in contrast to the course of CK, which increased until day 2 and 3 after BICU admission until its decrease. Thus, in contrast to myoglobin, CK on admission may not adequately reflect the severity of injury. One study on 37 HVI patients found that the CK serum concentration peaked on the second and third day following injury, whereas the highest CK measurements were obtained in patients with burn sizes of 21 to 40% TBSA. In contrast, patients with more extensive TBSA showed the highest CK levels on the day of admission, which subsequently decreased over the following days. In all patients with TBSA > 41%, serum levels decreased no later than the third day following injury and reached almost physiologic values within 1 week [18]. Other studies found a linear decrease of CK levels in all patients after HVI [19, 20]. To what extent the delay in CK increase is related to burn size or other factors is still debatable. However, the delay might be based on an enzyme-dependent activation characteristic, which is probably associated with latency in response to muscle tissue degeneration due to burns and cellular hydrops. Future studies should address this phenomenon to clarify underlying pathomechanisms. Moreover, it would be interesting to investigate first, whether rhabdomyolysis is influenced by early therapeutic measures (e.g., fasciotomy and/or burn wound excision) and second, whether different strategies of rhabdomyolysis therapy influence outcomes. The common treatment includes aggressive fluid resuscitation and adjuvant application of mannitol and bicarbonate for AKI prevention [21, 22]. However, randomized controlled trials regarding the benefit of both fluids and adjuvant pharmacological therapies are not available and clinical studies provided inconsistent results [21]. The role of continuous RRT remains unclear. In our patients, RRT was not used in an attempt to avoid AKI but only in cases of manifest AKI. A meta-analysis revealed insufficient evidence to discern any benefits of RRT over conventional therapy in the prevention of rhabdomyolysis-induced AKI [23]. In a small study on burn patients, myoglobin elimination myoglobin by RRT using a standard filter was not greater than that by fluid resuscitation alone [24]. However, some studies suggest the early use of newer membranes, such as AN69 ST150, to eliminate myoglobin [25, 26]. Recently, the application of novel cytokine adsorbers for myoglobin removal was proposed [27]. A prospective, randomized, non-blinded, controlled study on this topic is currently underway [28].

Although our data revealed that rhabdomyolysis was associated with the need for amputation and that amputation independently predicted mortality, other studies found that the need for amputation was not associated with increased mortality [29, 30]. Furthermore, one study found that the need for amputation was not associated with increasing burn size [9]. The reasons for these contradictory findings may be different patterns of injury, different thresholds and timings of amputation, and different infrastructures to perform limb salvage using microvascular tissue transplantation. The reliability of prognostic parameters after electrical injury should be interpreted with caution and in regard to the individual course and context of the case.

The current study has some strengths. Due to the long study period and the dual center design, we were able to include a relevant number of patients with reduced center-specific aspects. Our analysis allows us to form statements on the epidemiology and treatment of electrical injuries in a mid-European country. Many studies on electrical injuries have been conducted in considerably different health care systems, where the incidence, management and outcomes of electrical injuries may not be comparable [1]. The long study period and retrospective study design also represent a limitation of the current study. We cannot disclose whether therapy strategies were influenced by the implementation of new guidelines (e.g., introduction of low tidal volume ventilation in acute respiratory distress syndrome management, avoidance of fluid creep and secondary abdominal compartment syndrome in large burns, and improved sedation management) and/or changes in the departments’ administrative positions during the 18-year study period [31–34]. Furthermore, simultaneous concomitant injuries (e.g., falls from railway pylons) were not analyzed in this study, which could have influenced outcomes. However, although well-powered prospective randomized controlled studies in patients with electrical injuries would be desirable, there are many confounders that may not be addressed appropriately in these settings because of low incidence, incalculably high variability of current exposure, extension of burned TBSA, and predisposing morbidity.

## Conclusions

Our data consistently confirm the results of previous studies supporting the need for specialized burn intensive care after electrical injury. Particularly in HVI, life-threating complications may occur. A high proportion of these patients need complex surgical interventions (e.g., microvascular tissue transplantation). Serum myoglobin and CK levels reflect the severity of injury and may predict a more complex clinical course. The delayed onset of CK peak levels after HVI needs to be investigated in further studies. Routine cardiac monitoring > 24 h post injury does not seem to be necessary.

## Abbreviations

ABSI: Abbreviated burn severity index
AKI: Acute kidney injury
ARDS: Acute respiratory distress syndrome
BICU: Burn intensive care unit
BNP: Brain natriuretic peptide
CI: Confidence interval
CK: Creatine kinase
CPR: Cardiopulmonary resuscitation
CT: Computed tomography
CVVHD: Continuous veno-venous hemodialysis
CVVHDF: Continuous veno-venous hemodiafiltration
ED: Emergency department
EMS: Emergency medical service
HVI: High voltage injury
LOS: Length of stay
LVI: Low voltage injury
OR: Odds ratio
PEA: Pulseless electric activity
PID: Post injury day
RRT: Renal replacement therapy
SD: Standard deviation
TBSA: Total body surface area
